# Selected Pathological Criteria That Predict Low Rates of Axillary Lymph Node Metastases Regardless of Patient Age: A Single Institution Study

**DOI:** 10.1155/tbj/6196988

**Published:** 2026-04-11

**Authors:** S. David Nathanson, Shravan Leonard-Murali, Jenny Bui, Dhananjay A. Chitale, Jessica Bensenhaver, Theresa Schwartz, Lindsay Petersen, Kylie Springer, Patricia Baker, Laura L. Susick

**Affiliations:** ^1^ Department of Surgery, Henry Ford Health, Detroit, Michigan, USA, henryford.com; ^2^ Division of Surgical Oncology, Department of Surgery, Allegheny Health Network, Pittsburgh, Pennsylvania, USA, ahn.org; ^3^ Department of Pathology, Henry Ford Health, Detroit, Michigan, USA, henryford.com; ^4^ Department of Public Health Sciences, Henry Ford Health, Detroit, Michigan, USA, henryford.com

**Keywords:** age, breast cancer, lymphovascular invasion, sentinel lymph node metastases, tumor grade, tumor size

## Abstract

**Background and Aims:**

Omission of sentinel node biopsy is increasingly offered to selected older women with cN0 low‐risk breast cancer (BC). We hypothesized that some younger women might exhibit a low enough incidence of lymph node metastases to possibly justify excluding axillary surgery.

**Methods:**

We statistically analyzed, using parametric and nonparametric tests as appropriate, multiple demographic and clinicopathologic variables in cT1‐2 N0 M0 BC patients of all ages undergoing axillary LN excisional surgery from a long‐term, prospectively maintained database.

**Results:**

Patients with (816) and without (3617) LN metastases were compared. Although older patients were significantly (*p* < 0.0001) less likely to have LN metastases compared to younger patients, 3/61 (4.92%) of those < 50 years old with grade 1 tumors ≤ 1 cm in size (T1a and b) and no lymphovascular invasion had LN metastases compared to 30/504 (5.95%) ≥ 50. Patients aged 50 or older with Grade 2/3, < 1 cm, LVI‐negative tumors had only 53/774 (6.85%) LN positive, compared to 19/131 (14.5%) in women < 50 with the same pathology.

**Conclusions:**

Women with grade 1, ≤ 1 cm invasive BCs, and no LVI had < 6% incidence of LN metastases regardless of age. Instead of excluding younger women from axillary node surgery de‐escalation strategies, this study suggests that any woman with a tumor size ≤ 1 cm, Grade 1, and no LVI could be evaluated in prospective studies whose objective is to safely avoid axillary LN surgery.

**Synopsis**
 The goal of de‐escalating axillary surgery in BC patients is predicated upon age ≥ 50 years and other factors. This study demonstrates a select group of patients < 50 with a low incidence of lymph node metastases who might also benefit from omission of lymph node biopsy.

## 1. Introduction

Following Halsted’s introduction of the radical mastectomy for breast cancer (BC) in the late nineteenth century, almost all BC patients were advised to undergo complete axillary lymph node dissection (CALND), both for clinically confirmed lymph node (LN) metastases and also prophylactically in patients with clinically negative (cN0) axillae [1]. Since then axillary LN surgery has experienced 4 major phases. For at least 8 decades, Levels I, II, and III CALND was advised for all patients with invasive BC as part of the radical mastectomy. During the subsequent phase, only Levels I and II nodes were removed as part of the modified radical mastectomy [2] and, later, associated with lumpectomy to excise the primary BC. Level III LNs were not removed prophylactically. The next phase was the introduction of the sentinel LN biopsy (SLNB) [3, 4], and following the ACOSOG Z0011 study [5], selected patients with tumor‐positive SLNs have avoided CALND. We are currently in the fourth phase during which we select patients in whom both SLNB and CALND might be completely avoided.

Detailed pathologic information about the regional axillary LNs in BC provides important staging and prognostic insights, often crucial to assigning the correct management decisions guided by national guidelines such as the National Cancer Center Network (NCCN). However, there is increasing reliance on molecular/genetic markers in aiding clinicians in the choice of adjuvant therapies for BC [6] that might decrease, or even abrogate, the need to know the pathologic status of the SLN. Improved regional LN field control, where surgical removal of all microscopic and macroscopic disease, once thought to be vital for locoregional and systemic control of BC, is no longer thought essential in all cases (NCCN and American Society of Clinical Oncology guidelines) [7]. This contrasts with melanoma, where successful systemic treatments are not yet as effective as in BC and where SLNB is still selectively advocated [8]. The pathologic status of axillary SLNs in BC is also a requirement in many clinical trials.

While the morbidity associated with SLNB is relatively mild compared to CALND [8], seromas, infections, nerve damage, and lymphedema are occasionally encountered with this procedure. Another reason to consider eliminating SLNB is the cost of the operation, nuclear medicine, and pathology.

The ideal person in whom an SLNB might be avoided would be one with negative LNs. The problem is our ability to accurately determine the presence of LN metastases before the patient has any surgery on the primary BC. Clinical examination of the axilla has high false positive and false negative rates and is only useful in the clinical determination of axillary stage by the finding of obvious enlarged, usually firm, LNs with metastases. Axillary ultrasound is more accurate than clinical examination and has become useful in clinical practice [9]. The recently reported SOUND study from Europe [10] shows that it is safe to avoid SLNB in older women with BC under 2 cm in size where axillary ultrasound fails to show signs of metastasis. The false negative ultrasound rate in that study was 13.7%, with a locoregional recurrence rate of 1.6% in those patients who had no axillary surgery. The Society of Surgical Oncology ‘Choosing Wisely’ guidelines [11] allow us to consider avoiding removal of any ipsilateral axillary LNs in otherwise healthy women aged above 70 years with low‐risk BC. In those older patients the incidence of regional LN metastasis may be quite low.

Because of the conclusions in the SOUND study and the ‘Choosing Wisely’ guidelines, with a focus on age, the study presented here was designed to investigate the incidence of SLN metastases in older compared to younger BC patients with the idea of identifying a possible cut‐off age where the frequency of LN metastases might be low enough to justify omission of SLNB. We incorporated other clinicopathological variables possibly associated with LN metastases in a large single‐institution database of early BC patients to determine which pathologic variables plus age could more accurately identify patients with a low enough incidence of LN metastases to conceivably justify omission of SLNB.

## 2. Methods

### 2.1. Patients

Female patients with cT1‐2 N0 M0 BC undergoing SLNB within our quaternary care hospital system from 1995 through 2022 were included in this study. The BC patients were diagnosed and treated by the Henry Ford Breast Cancer Services, a highly specialized, guideline‐controlled, multidisciplinary team of BC physicians and ancillary staff in a vertically integrated academic health system, approved by an American College of Surgeons site visit every 3 years. The multidisciplinary breast team management of every BC patient was tracked, reported, and audited during twice‐weekly BC tumor boards and clinics. NCCN guidelines were used in managing all patients.

Excluded from this study were patients who were men, had prior chemotherapy or breast irradiation, had pure ductal carcinoma in situ (DCIS) or lobular carcinoma in situ (LCIS) without invasion, or were missing pathologic information. Adjuvant systemic therapy and radiotherapy are not included since those postoperative treatment modalities do not influence lymph node metastasis in our study.

### 2.2. Surgery and Pathologic Evaluation of Lymph Nodes and Primary Breast Tumors

Full‐time board‐certified surgeons, trained and competent in sentinel node surgery, did all the breast and axillary node surgery at the main Henry Ford Health Detroit main hospital campus and two affiliated Southeast Michigan suburban operating rooms as previously described [12]. During the initial learning phase of SLNB, when find rates were about 90%, a complete level 1 and 2 axillary lymphadenectomy was done in cases whether the surgeon found the SLN or not. Histologic LN positivity was either ‘yes’ or ‘no’ and recorded as such. Since the reporting of the ACOSOG Z0011 clinical trial [5], CALND has been omitted in most cases even when the SLN was positive. In cases where a lumpectomy was done for DCIS without SLNB, a second operation to excise the SLN was done if the pathologist found invasive components in the DCIS lumpectomy specimen. DCIS cases were excluded from analysis if invasion was not found where a mastectomy or lumpectomy with SLNB for DCIS had been done.

Pathologic evaluation of excised LNs and primary breast tumors was undertaken by fellowship‐trained breast pathologists. Processing of SLNs was accomplished using the College of American Pathologists LN protocol and American Society of Clinical Oncology guidelines [13]. Briefly, the SLN was dissected from surrounding perinodal fat and serially sectioned along the short axis (2‐3 mm slices). Tissues were processed using a standard tissue processing protocol. The formalin‐fixed paraffin‐embedded blocks were sectioned at 4‐5 microns and stained with hematoxylin and eosin. The largest metastatic deposit in the first SLN identified was measured microscopically and reported as ‘positive’ whenever tumor cells were identified in the node.

### 2.3. Database Maintenance

Prospectively and continually collected data from all patients with invasive BC with cN0 ipsilateral axillae and no initial evidence of systemic disease, who had SLNB alone or SLNB plus CALND, were consistently audited and abstracted by the same collection team for 27 years and approved annually by the Institutional Review Board.

### 2.4. Statistical Analysis

From our database, established to include all early BC patients with cN0 ipsilateral axillae and no systemic metastases, the initial outcome of interest was the association of patient age with LN metastases. Included were BC patients aged 21 through 101 years old, evaluated by continuous age or grouped by selected cut‐off ages, or by decile. All continuous data are reported using mean, standard deviation, count, median, interquartile range (IQR), and range, while categorical data are reported as counts and percentages (*N* [%]). Univariate two‐group comparisons were performed using a t‐test for normally distributed continuous variables, Wilcoxon Rank Sum for skewed continuous variables and chi‐square or Fisher’s test if cell counts were < 5 for categorical variables. The variables assessed included age, race, hormone receptor status (estrogen receptor [ER] and progesterone receptor [PR]), HER2/neu expression, triple‐negative BC, LVI, tumor size (T), location in the breast, grade, LN (N) stage at diagnosis, and overall stage at diagnosis. Univariate and multivariable logistic regression models were utilized to estimate crude and adjusted odds ratios (ORs) and 95% confidence intervals (95% CIs) for associations between predictors listed above and LN metastases. Our study assessed 6 forced models, which included the variables listed above with specific control for redundancy depending on the model. The variables grouped together and analyzed were the following: (a) tumor size, LVI, age, and grade; (b) tumor size, LVI, age, and triple negative (ER, PR, and HER2/neu negative); (c) tumor size, LVI, age, HER2/neu, ER, and PR; (d) tumor size, LVI, age, HER2/neu, ER, PR, and race; (e) tumor size, LVI, age, HER2/neu, ER, PR, and triple negative; and (f) tumor size, LVI, age, HER2/neu, triple negative, and grade.

Patients were grouped according to age (< 50, ≥ 50 to < 70, and ≥ 70) and selected combinations of tumor size (≤ 1, 1 to ≤ 2, and > 2), grade (1 or 2/3) and LVI (present or absent). Statistical significance was established at *p* < 0.05. All statistical analyses were performed in SAS 9.4 (SAS Institute, Cary, NC).

Artificial intelligence‐generated content (AIGC) tools were avoided in writing this manuscript.

## 3. Results

There were a total of 4988 patients in the database. Five hundred fifty‐five (11.1%) patients were excluded: men (*n* = 40); missing T size (*n* = 209); patients undergoing neoadjuvant chemotherapy (*n* = 284); and missing LN information (*n* = 22). One thousand five hundred sixty‐nine (35.4%) of 4433 patients included had primary tumors ≤ 1 cm in size (T1a = 504; T1b = 1065). The average age of those patients included in the study was 62.37 ± 12.58 years old spanning from 21 to 101 years old. Patients with LN metastases (816; 18.4%) were on average younger than those without LN metastases (60.46 vs 62.80 years old, respectively; *p* < 0.0001) (Table 1). Patients with LN metastases were more likely to express positive HER2/neu, had on average a larger tumor size, and were more likely to have a Grade 2 or Grade 3 tumor and positive LVI.

**TABLE 1:** Descriptive statistics and two group comparisons.

	Lymph node metastasis			*p* value
	Total (*N* = 4433)	No (*N* = 3617)	Yes (*N* = 816)	
Age				< 0.0001
*N*	4433	3617	816	
Mean (SD)	62.37 (12.58)	62.80 (12.44)	60.46 (13.01)	
Median (IQR)	63.00 (53.00, 72.00)	63.00 (54.00, 72.00)	60.00 (51.00, 71.00)	
Range	21.00, 101.00	21.00, 101.00	24.00, 94.00	
Age group, *n* (%)				< 0.0001
< 60	1859 (41.9%)	1459 (40.3%)	400 (49.0%)	
≥ 60	2574 (58.1%)	2158 (59.7%)	416 (51.0%)	
Age group, *n* (%)				< 0.0001
< 50	754 (17.0%)	572 (15.8%)	182 (22.3%)	
> 50	3679 (83.0%)	3045 (84.2%)	634 (77.7%)	
Age group, *n* (%)				0.0251
< 40	136 (3.1%)	101 (2.8%)	35 (4.3%)	
≥ 40	4297 (96.9%)	3516 (97.2%)	781 (95.7%)	
Age group, *n* (%)				0.0017
< 45	363 (8.2%)	274 (7.6%)	89 (10.9%)	
≥ 45	4070 (91.8%)	3343 (92.4%)	727 (89.1%)	
Race, *n* (%)				0.7439
Black	1441 (34.0%)	1184 (34.2%)	257 (32.8%)	
Other	200 (4.7%)	163 (4.7%)	37 (4.7%)	
White	2599 (61.3%)	2110 (61.0%)	489 (62.5%)	
Missing	193	160	33	
ER, *n* (%)				0.1731
Negative	781 (17.8%)	650 (18.2%)	131 (16.2%)	
Positive	3600 (82.2%)	2921 (81.8%)	679 (83.8%)	
Missing	52	46	6	
PR, *n* (%)				0.0793
Negative	1112 (25.2%)	926 (25.8%)	186 (22.8%)	
Positive	3295 (74.8%)	2666 (74.2%)	629 (77.2%)	
Missing	26	25	1	
HER2/neu, *n* (%)				0.0007
Negative	3260 (83.3%)	2666 (84.3%)	594 (79.1%)	
Positive	655 (16.7%)	498 (15.7%)	157 (20.9%)	
Missing	518	453	65	
Triple negative, *n* (%)				0.0589
No	3966 (89.5%)	3221 (89.1%)	745 (91.3%)	
Yes	467 (10.5%)	396 (10.9%)	71 (8.7%)	
Tumor size				< 0.0001
*N*	4433	3617	816	
Mean (SD)	1.62 (1.05)	1.52 (1.02)	2.06 (1.08)	
Median (IQR)	1.40 (0.90, 2.10)	1.30 (0.80, 2.00)	1.80 (1.20, 2.60)	
Range	0.00, 5.00	0.00, 5.00	0.01, 5.00	
Tumor size grouping, *n* (%)				< 0.0001
T1b	1065 (32.4%)	978 (34.7%)	87 (18.4%)	
T1a	504 (15.3%)	465 (16.5%)	39 (8.3%)	
10–20 mm	1723 (52.3%)	1377 (48.8%)	346 (73.3%)	
Missing	1141	797	344	
Grade, *n* (%)				< 0.0001
G1	1098 (25.0%)	969 (27.0%)	129 (15.9%)	
G2/3	3296 (75.0%)	2615 (73.0%)	681 (84.1%)	
Missing	39	33	6	
Location, *n* (%)				0.0511
Central	140 (3.2%)	108 (3.0%)	32 (4.0%)	
LIQ	524 (12.0%)	418 (11.7%)	106 (13.3%)	
LOQ	516 (11.8%)	411 (11.5%)	105 (13.2%)	
UIQ	974 (22.3%)	822 (23.1%)	152 (19.0%)	
UOQ	2209 (50.6%)	1806 (50.7%)	403 (50.5%)	
Missing	70	52	18	
LVI, *n* (%)				< 0.0001
Negative	3860 (87.1%)	3321 (91.8%)	539 (66.1%)	
Positive	573 (12.9%)	296 (8.2%)	277 (33.9%)	
Mastectomy, *n* (%)				< 0.0001
No	3124 (70.6%)	2615 (72.4%)	509 (62.5%)	
Yes	1303 (29.4%)	997 (27.6%)	306 (37.5%)	
Missing	6	5	1	

Abbreviations: ER, estrogen receptor; IQR, interquartile range; LVI, lymphovascular invasion; PR, progesterone receptor; SD, standard deviation.

For every 10 year increase in age, the odds of having LN metastases were lower (Table 2). Patients that were < 40 years old were 1.56 (95% CI 1.05–2.31) times more likely to have LN metastases as compared to patients 40 or older. Patients that were < 45 years old were 1.49 (95% CI 1.16–1.92) times more likely to have LN metastases as compared to patients 45 or older. Patients that were < 50 years old were 1.53 (95% CI 1.27–1.84) times more likely to have LN metastases as compared to patients 50 or older. Patients that were < 60 years old were 1.42 (95% CI 1.22–1.66) times more likely to have LN metastases as compared to patients 60 or older. Patients with Grade 1 tumors were 0.51 (95% CI 0.42–0.63) times less likely to have LN metastases as compared to those with Grades 2/3. Patients with LVI were 5.77 (95% CI 4.78–6.96) times more likely to have LN metastases as compared to those without LVI. Patients whose tumors expressed a positive HER2/neu were 1.41 (95% CI 1.16–1.73) times more likely to have LN metastases as compared to those with a negative HER2/neu. For every 1 cm increase in primary breast tumor size, the odds of having LN metastases were 1.56 (95% CI 1.46–1.67) times more likely.

**TABLE 2:** Univariate odds ratios of LN metastases vs no LN metastases.

Covariate	Level	*N*	Lymph node meta = yes	OR *p* value
			Odds ratio (95% CI)	
Age (10 year change)		4433	0.86 (0.81–0.92)	< 0.001

Age group 50	< 50	754	1.53 (1.27–1.84)	< 0.001
	≥ 50	3679	—	—

Age group 60	< 60	1859	1.42 (1.22–1.66)	< 0.001
	≥ 60	2574	—	—

Age group	20–30	17	0.55 (0.14–2.16)	0.395
	30–40	119	0.63 (0.26–1.52)	0.307
	40–50	618	0.56 (0.25–1.24)	0.155
	50–60	1105	0.44 (0.20–0.97)	0.042
	60–70	1186	0.36 (0.16–0.78)	0.010
	70–80	1006	0.34 (0.15–0.74)	0.007
	80–90	354	0.31 (0.14–0.71)	0.006
	90–100+	28	—	—

Race	Black	1441	0.94 (0.79–1.11)	0.442
	Other	200	0.98 (0.68–1.42)	0.913
	White	2599	—	—

ER	Positive	3600	1.15 (0.94–1.41)	0.173
	Negative	781	—	—

PR	Positive	3295	1.18 (0.98–1.41)	0.080
	Negative	1112	—	—

HER2/neu	Positive	655	1.42 (1.16–1.73)	< 0.001
	Negative	3260	—	—

Triple negative	Yes	467	0.78 (0.60–1.01)	0.060
	No	3966	—	—

Grade	G1	1098	0.51 (0.42–0.63)	< 0.001
	G2/3	3296	—	—

Location	Central	140	1.33 (0.88–2.00)	0.174
	LIQ	524	1.14 (0.89–1.44)	0.294
	LOQ	516	1.14 (0.90–1.46)	0.269
	UIQ	974	0.83 (0.68–1.02)	0.071
	UOQ	2209	—	—

LVI	Positive	573	5.77 (4.78–6.96)	< 0.001
	Negative	3860	—	—

Tumor Size		4433	1.56 (1.46–1.67)	< 0.001

Abbreviations: ER, estrogen receptor; LVI, lymphovascular invasion; OR, odds ratio; PR, progesterone receptor.

Every 10 year increase in age is associated with lower odds of LN metastases when adjusted for HER2/neu, tumor size, and LVI (Table 3). Every 1 cm increase in tumor size is associated with higher odds of LN metastases when adjusted for age, HER2/neu, and LVI. LVI is associated with higher odds of LN metastases when adjusted for age, HER2/neu, and tumor size. Patients younger than 50 years old are more likely to have LN metastases when adjusted for HER2/neu, tumor size, and LVI (Table 4). Other multivariable analyses (see Methods) consistently revealed older age, smaller tumor size, absent LVI, and lower grade as statistically significant factors associated with decreased LN metastases (*p* < 0.0001).

**TABLE 3:** Multivariable logistic regression model with continuous age.

Effect	Odds ratio estimate	95% confidence limits		*p* value
Age (10 year change)	0.878	0.821	0.939	0.0001
HER2 (positive vs negative)	1.166	0.938	1.449	0.1661
Tumor size	1.564	1.444	1.694	< 0.0001
LVI (positive vs negative)	4.593	3.755	5.619	< 0.0001

Abbreviation: LVI, lymphovascular invasion.

**TABLE 4:** Multivariable logistic regression model with age cut‐off of 50 used.

Effect	Odds ratio estimate	95% confidence limits		*p*‐value
Age < 50 vs ≥ 50	1.413	1.141	1.749	0.0015
HER2 (positive vs negative)	1.179	0.949	1.465	0.1361
Tumor size	1.561	1.441	1.690	< 0.0001
LVI (positive vs negative)	4.604	3.764	5.631	< 0.0001

Abbreviation: LVI, lymphovascular invasion.

Table 5 shows a low (30/504; 5.95%) incidence of SLN metastasis in women 50 years old and older when coupled with tumor sizes of 1 cm or less, grade 1, and no LVI. Patients ≥ 50 years old with grade 2/3 tumors, ≤ 1 cm and negative LVI also had a low incidence (53/774; 6.85%) of SLN metastases. Of 61 women age < 50 years old with grade 1, ≤ 1 cm, and LVI negative, only 3 (4.92%) had SLN metastases, while 19/131 (14.5%) with grade 2/3 tumors, ≤ 1 cm and LVI negative had SLN metastases.

**TABLE 5:** Likelihood of axillary node metastases related to LVI, age, grade, and primary tumor size.

Age	Size (cm)	Grade	LVI	LN negative *n* (%)	LN positive *n* (%)
≥ 50	≤ 1	G1	Negative	474 (94.05%)	30 (5.95%)
		G1	Positive	9 (81.82%)	2 (18.18%)
	> 1	G1	Negative	356 (83.57%)	70 (16.43%)
		G1	Positive	11 (55%)	9 (45%)
	≤ 1	G2/3	Negative	721 (93.15%)	53 (6.85%)
		G2/3	Positive	40 (78.43%)	11 (21.57%)
	> 1	G2/3	Negative	1224 (82.09%)	267 (17.91%)
		G2/3	Positive	178 (48.90%)	186 (51.10%)
	> 1, ≤ 2	G1	Negative	269 (85.13%)	47 (14.87%)
		G1	Positive	8 (57.14%)	6 (42.86%)
	> 1, ≤ 2	G2/3	Negative	754 (83.78%)	146 (16.22%)
		G2/3	Positive	102 (56.98%)	77 (43.02%)
	> 2	G1	Negative	87 (79.09%)	23 (20.91%)
		G1	Positive	3 (50%)	3 (50%)
	> 2	G2/3	Negative	470 (79.53%)	121 (20.47%)
		G2/3	Positive	76 (41.08%)	109 (58.92%)

< 50	≤ 1	G1	Negative	58 (95.08%)	3 (4.92%)
		G1	Positive	0 (0%)	1 (100%)
	> 1	G1	Negative	59 (83.10%)	12 (16.90%)
		G1	Positive	2 (50%)	2 (50%)
	≤ 1	G2/3	Negative	112 (85.50%)	19 (14.50%)
		G2/3	Positive	6 (60%)	4 (40%)
	> 1	G2/3	Negative	286 (78.14%)	80 (21.86%)
		G2/3	Positive	48 (44.04%)	61 (55.96%)
	> 1, ≤ 2	G1	Negative	49 (89.09%)	6 (10.91%)
		G1	Positive	2 (50%)	2 (50%)
	> 1, < 2	G2/3	Negative	160 (79.60%)	41 (20.40%)
		G2/3	Positive	29 (59.18%)	20 (40.82%)
	> 2	G1	Negative	10 (62.50%)	6 (37.50%)
		G1	Positive	n/a	n/a
	> 2	G2/3	Negative	126 (76.36%)	39 (23.64%)
		G2/3	Positive	19 (31.67%)	41 (68.33%)

≥ 70	≤ 1	G1	Negative	199 (94.31%)	12 (5.69%)
		G1	Positive	3 (100%)	0 (0%)
	> 1	G1	Negative	149 (88.17%)	20 (11.83%)
		G1	Positive	1 (20%)	4 (80%)
	≤ 1	G2/3	Negative	257 (93.80%)	17 (6.20%)
		G2/3	Positive	13 (81.25%)	3 (18.75%)
	> 1	G2/3	Negative	468 (82.69%)	98 (17.31%)
		G2/3	Positive	66 (51.56%)	62 (48.44%)
	> 1, ≤ 2	G1	Negative	109 (88.62%)	14 (11.38%)
		G1	Positive	0 (0%)	2 (100%)
	> 1, ≤ 2	G2/3	Negative	296 (82.45%)	63 (17.55%)
		G2/3	Positive	42 (59.15%)	29 (40.85%)
	> 2	G1	Negative	40 (86.96%)	6 (13.04%)
		G1	Positive	1 (33.33%)	2 (66.67%)
	> 2	G2/3	Negative	172 (83.09%)	35 (16.91%)
		G2/3	Positive	24 (42.11%)	33 (57.89%)

< 70	≤ 1	G1	Negative	333 (94.07%)	21 (5.93%)
		G1	Positive	6 (66.67%)	3 (33.33%)
	> 1	G1	Negative	266 (81.10%)	62 (18.90%)
		G1	Positive	12 (63.16%)	7 (36.84%)
	≤ 1	G2/3	Negative	576 (91.28%)	55 (8.72%)
		G2/3	Positive	33 (73.33%)	12 (26.67%)
	> 1	G2/3	Negative	1042 (80.71%)	249 (19.29%)
		G2/3	Positive	160 (46.38%)	185 (53.62%)
	> 1, ≤ 2	G1	Negative	209 (84.27%)	39 (15.73%)
		G1	Positive	10 (62.50%)	6 (37.50%)
	> 1, ≤ 2	G2/3	Negative	618 (83.29%)	124 (16.71%)
		G2/3	Positive	89 (55.69%)	68 (43.31%)
	> 2	G1	Negative	57 (71.25%)	23 (28.75%)
		G1	Positive	2 (66.67%)	1 (33.33%)
	> 2	G2/3	Negative	424 (77.23%)	125 (22.77%)
		G2/3	Positive	71 (37.77%)	117 (62.23)

≥ 50, < 70	≤ 1	G1	Negative	275 (93.86%)	18 (6.14%)
		G1	Positive	6 (75%)	2 (25%)
	> 1	G1	Negative	207 (80.54%)	50 (19.46%)
		G1	Positive	10 (66.67%)	5 (33.33%)
	≤ 1	G2/3	Negative	464 (92.80%)	36 (7.20%)
		G2/3	Positive	27 (77.14%)	8 (22.86%)
	> 1	G2/3	Negative	756 (81.73%)	169 (18.27%)
		G2/3	Positive	112 (47.46%)	124 (52.54%)
	> 1, < 2	G1	Negative	160 (82.90%)	33 (17.10%)
		G1	Positive	8 (66.67%)	4 (33.33%)
	> 1, < 2	G2/3	Negative	458 (84.66%)	83 (15.34%)
		G2/3	Positive	60 (55.56%)	48 (44.44%)
	> 2	G1	Negative	46 (73.44%)	17 (26.56%)
		G1	Positive	2 (66.67%)	1 (33.33%)
	> 2	G2/3	Negative	298 (77.60%)	86 (22.40%)
		G2/3	Positive	52 (40.63%)	76 (59.38%)

*Note:* n/a–no patients seen with the combination.

## 4. Discussion

Despite changes in BC management during 27 years of data accumulation, determination of the pathologic presence of LN metastasis by surgical LN excision has remained the same. Multivariate analysis allowed us to determine which combination of pathologic and demographic variables affect the likelihood of LN metastasis. Our data show that primary tumor size, grade, LVI status, and age, alone or in combination, affect the incidence of ipsilateral axillary BC metastases. When filtered and analyzed according to those four variables, we found a wide range of LN metastasis incidences. Women of all ages with 1 cm or smaller, low‐grade primary BCs and no LVI had < 6% chance of having LN metastases. In addition, patients aged 50 years old or older (but not younger than 50) had < 7% of LN metastases even with higher grade (2 or 3) BCs if the tumors did not exhibit LVI and were smaller than 1 cm. The tendency for patients with tumors expressing HER2/neu to have more LN metastases in univariate analysis was not a significant factor in the multivariable analyses. Similarly, hormone receptor expression and race were not significantly associated with LN metastases. Patients with triple‐negative BC showed a significantly lower incidence of LN metastases. Mastectomy was often chosen in the management of larger and more aggressive BCs, which may account for a slightly higher incidence of LN metastases with this procedure compared to lumpectomy.

Primary tumor size is significantly predictive of LN metastases in BC [14], melanoma [15], and some animal [16] malignancies. Size alone and sometimes combined with other clinical and pathological criteria [17–19] has long been known to be associated with the incidence of LN metastases. Some of these studies have suggested that the larger, the tumor the more likely LVI.

Histological grade can predict tumor behavior [20]. An important determinant of BC outcome [21], grade provides equivalent predictive information to that of LN status [22] and greater than that of tumor size in predicting the likelihood of systemic metastases [23]. Higher tumor grade, associated with an increased rate of proliferation of tumor cells, is often associated with an increased likelihood of LN metastases [24]. The reasons why higher‐grade tumors are more likely to metastasize to LNs are not clear, but it is possible that the genetic machinery important to metastases is more likely to be altered in higher‐grade cells [25].

The incidence of axillary LN metastases in BC has been reported to be less in older patients. The reasons for this phenomenon might be associated with the mechanical decline of aging lymphatics. The process of gradual physiological deterioration affects lymphatic efficiency like other age‐dependent decreases in physiological functions [26]. Aging induces structural and functional changes in lymphatic networks [27]. Decreased lymphatic contractility, with resultant decreased pumping efficiency, can result in interstitial tissue space fluid retention. Although the relative inadequacy causes primary functional impediments to fluid and cell uptake in peripheral lymphatic vessels, it is not clear whether this age‐related inefficiency affects the incidence of axillary LN metastases. In our study older women did not have observably less aggressive tumors to account for the significantly lower likelihood of LN metastases. Older patients undergoing SLNB have lower intraoperative radio‐colloid gamma probe counts, suggesting lower lymphatic flow rates [28]. The lower lymphatic flow rates in older women [26] might also translate to lower mechanical efficiency in tumor cell transport; fewer tumor cells transported in lymphatic trunks per unit time [29] could decrease the incidence of LN metastases in older women.

The probability of dying from BC in women 70 years and older treated surgically with tumors that are hormone‐receptor positive and HER2/neu negative is about 1% [30]. Older age women are also more likely to have comorbidities and associated complications from locoregional and systemic treatments. In a carefully controlled randomized study comparing lumpectomy with or without adjuvant irradiation, with or without SLNB, there were no survival benefits from SLNB [31]. Axillary staging with SLNB in selected women ≥ 70 years is therefore no longer routinely recommended [32], although implementation of this new policy has been difficult [33]. The data reported in our study may further justify avoiding SLNB in many women ≥ 70 years old with small, low‐grade BCs without LVI.

It is mechanically logical that LVI is a strong predictor of LN metastases in our study. Invasion of new and existing peri‐ or intratumoral lymphatics is accomplished by metastatically aggressive tumor cells [34]. Such tumor cells invading the lymphatic capillaries in, and around primary BCs are seen on hematoxylin and eosin‐stained slides as single or clumped cells within an endothelial‐lined space and recorded as ‘LVI positive’ by pathologists. To reach the SLN, the intralymphatic tumor cells flow proximally through lymphatic trunks to enter the subcapsular sinuses of SLNs through afferent lymphatics. Small numbers of subcapsular tumor cells may be missed on microscopic evaluation and it may take time for those cells to invade the cortical parenchyma and grow large enough to be visible and be recorded as ‘LN positive.’ The accuracy of the pathological observation of both LVI and LN positivity is highly dependent upon sampling precision, experience, and the use of ancillary techniques such as immunohistochemistry to highlight blood vessel and lymphatic endothelial cells, especially if LVI is not seen when the SLN shows metastases. Therefore, the incidence of LVI and LN positivity is probably higher than observed in our cases. Specific lymphatic endothelial cell immunohistochemical staining improves the observation of true lymphatic versus blood vessel invasion [35]. LNs may be metastases‐free when LVI is positive, since intralymphatic tumor cells may not yet have reached the LN at the time of sampling. LNs falsely negative microscopically may be positive for metastases when examined using immunohistochemistry and biologic assays [36]. The missing pathology in our study might have added information, although it would likely not have altered our conclusions.

LVI is best evaluated by looking at the entire tumor and is less accurate when evaluating needle biopsies. Therefore, the appropriate clinical use of LVI to predict which patients to omit SLNB will require new ideas to implement further surgical axillary management. One example might be to do a lumpectomy alone without SLNB in patients with small, low‐grade tumors, followed by a decision about axillary management in the small number of patients found to have LVI.

Omission of axillary LN surgery is already practiced for some older women with BC. Our study suggests that younger women below 50 years of age might also be considered for de‐escalation of sentinel node biopsy if their primary tumors are of low grade, show no evidence of LVI, and are smaller than 1 cm in diameter. Such a change in surgical practice would require a future clinical study in which younger patients with these pathologic and demographic criteria are prospectively and randomly selected for entry and their outcome compared.

## Funding

This study was funded by the Nathanson/Rands Chair in Breast Cancer Research and the Team Angels Organization of Michigan.

## Disclosure

Previous presentation was presented at the 25^th^ Annual Meeting of the American Society of Breast Surgeons, Orlando, Florida, April 2024. The paper and its contents were edited by Stephanie Stebens MLIS, AHIP, Librarian, Henry Ford Health, Detroit, Michigan. The financial sources had no role in the study design, collection, analysis, interpretation of data, writing the report, or the decision to submit the manuscript for publication. The lead author, S. David Nathanson, affirms that this manuscript is an honest, accurate, and transparent account of the study being reported; that no important aspects of the study have been omitted; and that any discrepancies from the study as planned have been explained.

All authors have read and approved the final version of the manuscript. S. David Nathanson as corresponding author had full access to all of the data in this study and takes complete responsibility for the integrity of the data and the accuracy of the data analysis.

## Conflicts of Interest

The authors declare no conflicts of interest.

## Data Availability

The authors confirm that the data supporting the findings of this study are available within the article.
